# First person – Katherine Robinson

**DOI:** 10.1242/dmm.049289

**Published:** 2021-10-11

## Abstract

First Person is a series of interviews with the first authors of a selection of papers published in Disease Models & Mechanisms, helping early-career researchers promote themselves alongside their papers. Katherine Robinson is first author on ‘ Flow cytometry allows rapid detection of protein aggregates in cellular and zebrafish models of spinocerebellar ataxia 3’, published in DMM. Katherine is a PhD student in the lab of Dr Angela Laird at Macquarie University, Sydney, Australia, using pre-clinical animal models (transgenic zebrafish and mice) to find new therapeutics for the treatment of neurodegenerative diseases.


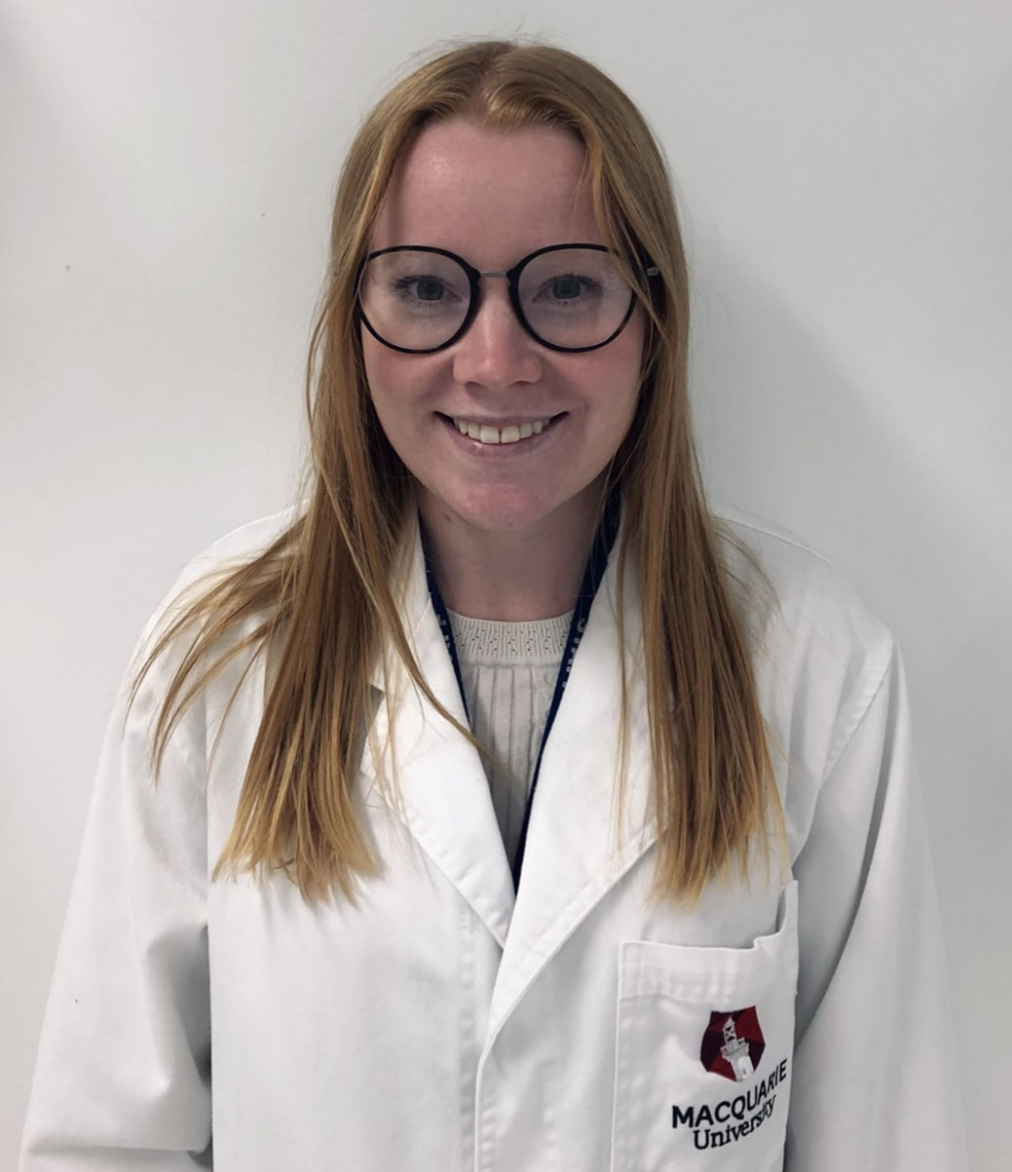


**Katherine Robinson**

**How would you explain the main findings of your paper to non-scientific family and friends?**

Spinocerebellar ataxia type 3 (SCA3, also known as Machado–Joseph disease) is a hereditary disease caused by inheritance of a mutation in a particular gene*.* Individuals that inherit the mutated form of this gene will develop movement problems, such as impaired balance, loss of coordination and wobbly gait (known as ataxia). SCA3 causes degeneration and death of neurons (nerve cells) involved in movement, eventually causing wheelchair dependence and mortality in patients. Sadly, there are no available treatments to prevent disease development or prolong life. One factor that is thought to contribute to the death of these neurons is the abnormal accumulation of proteins into protein clumps, known as protein aggregates. Protein aggregates can be detrimental to neurons, as they cannot be easily broken down or removed by the normal processes responsible for degrading and clearing cellular components. In this study, we used human cells and zebrafish carrying the mutant gene found in SCA3 patients. Next, we counted the number of protein aggregates using flow cytometry, a technique that analyses single cells or particles suspended in a solution as they flow past a laser. This is the first time that flow cytometry has been used to count protein aggregates in zebrafish, and the first time that flow cytometry has been used to examine protein aggregates in SCA3.

**What are the potential implications of these results for your field of research?**

In this study, we adapted a flow cytometric approach that allowed rapid identification of fluorescently tagged ataxin-3 protein aggregates in cultured cells and transgenic zebrafish. This approach had previously been explored for detecting protein aggregates in cell models of motor neuron disease or Huntington's disease; however, this was the first time this approach had been utilised for SCA3. Further, we expanded the approach and developed a flow cytometric method of examining protein aggregates in cells obtained from transgenic zebrafish expressing fluorescent proteins. We conclude that flow cytometric analysis of SCA3 cells and zebrafish presents a rapid, high-throughput read-out of protein aggregation and could be used in the future to complement screening of new treatments that aim to increase the clearance of ataxin-3 protein aggregates, thus reducing neuronal dysfunction and death.

**Figure:**
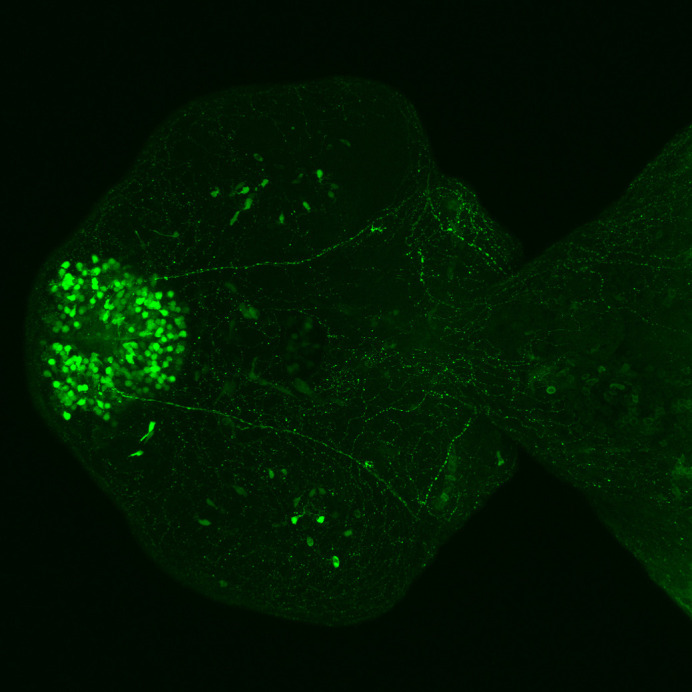
Microscopy image of SCA3 transgenic zebrafish brain showing expression of mutant human ataxin-3 protein fused to a green fluorescent protein within neurons.

**What are the main advantages and drawbacks of the model system you have used as it relates to the disease you are investigating?**

In this study, we utilised human neuroblastoma (SHSY5Y) cells and transgenic zebrafish overexpressing human ATXN3 fused to green fluorescent protein. One advantage of these models is that ataxin-3 protein aggregates can be visualised without the need for immunohistochemistry. A disadvantage of using cultured neuroblastoma cells is that these cells are both neuronal-like and cancer-like. Working with transgenic zebrafish models of neurodegenerative diseases has many advantages. Firstly, adult zebrafish can lay hundreds of embryos per mating, meaning we can experiment with hundreds of genetically identical animals each week. Secondly, zebrafish develop rapidly, and they can be easily manipulated to express genes that cause neurodegenerative diseases in humans, producing disease phenotypes akin to human disease within a matter of days. Lastly, zebrafish are well suited to drug-testing studies, as drugs can be added to raising media and readily absorbed. However, one drawback of the flow cytometric approach described in our study is that it would be challenging to adapt to adult zebrafish.

“[…] the most significant challenge to the field of neurodegenerative disease research is overcoming the barriers that prevent translation from efficacy in pre-clinical animal models to improvements for clinical populations.”

**What has surprised you the most while conducting your research?**

I am always pleasantly surprised by the versatility of zebrafish as a disease model! When embarking on this project, I was also surprised at the ease and relative speed of the method we adapted. We were able to take an entire zebrafish larva and break it down into single cells within an hour, delivering relatively fast and reproducible results!

**Describe what you think is the most significant challenge impacting your research at this time and how will this be addressed over the next 10 years?**

While we have known the genetic cause of SCA3 since the 1990s, there are currently no available treatments that can stop or slow the development of disease in patients that have inherited a mutated form of the *ATXN3* gene. Unfortunately, many of the new treatments and compounds that are found to be effective in pre-clinical animal studies do not yield significant improvements when trialled in patients with neurodegenerative diseases (of course, this is not only specific to neurodegenerative diseases!). This failure to translate findings from pre-clinical animal models to significant improvements in humans can be due to a range of reasons including issues with safety and toxicity, lack of specificity, failure to cross the blood brain barrier and lack of understanding relating to the mechanism of action. I believe that the most significant challenge to the field of neurodegenerative disease research is overcoming the barriers that prevent translation from efficacy in pre-clinical animal models to improvements for clinical populations.

“The culture of academia can disproportionately affect childbearing early-career scientists […]”

**What changes do you think could improve the professional lives of early-career scientists?**

The culture of academia can disproportionately affect childbearing early-career scientists as periods of parental leave can have a negative effect on research progress, leaving substantial gaps in your publication record, making individuals less competitive for future employment and grant funding. I believe that more can be done to support scientists returning to research after periods of parental leave. The development of additional funding schemes and fellowships dedicated to supporting individuals who are wanting to start a family, or those returning to academia after periods of parental leave, would go a long way to improving the lives of early-career scientists.

**What's next for you?**

Our team has recently utilised the transgenic zebrafish model and the flow cytometry approach described in this paper to test a novel compound that inhibits the activity of calpains, enzymes that can break proteins down into smaller protein fragments. We compared this new compound to calpeptin, the calpain inhibitor compound used in this study, and found that treatment with a novel calpain inhibitor can also yield protective effects in our transgenic SCA3 zebrafish model. We are excited to progress these compounds to the next stage of our drug-testing pipeline and hope that they may slow or halt disease progression in patients in the future!
